# A Genetic Tool for Non‐Invasive Monitoring of Fennoscandian Golden Eagles (*Aquila chrysaetos*)

**DOI:** 10.1002/ece3.74386

**Published:** 2026-09-20

**Authors:** Benjamin Lindberg, Oddmund Kleven, Laura Kvist, Anita Norman, Helena Köningsson, Karl‐Otto Jacobsen, Per‐Olof Nilsson, Navinder J. Singh, Göran Spong

**Affiliations:** ^1^ Department of Wildlife, Fish and Environmental Studies, Faculty of Forest Sciences Swedish University of Agricultural Sciences Umeå Sweden; ^2^ Norwegian Institute for Nature Research (NINA) Trondheim Norway; ^3^ Ecology and Genetics Research Unit University of Oulu Oulu Finland; ^4^ Norwegian Institute for Nature Research (NINA) Tromsø Norway

**Keywords:** genetic monitoring, genotyping, population, single nucleotide polymorphism, SNP markers

## Abstract

Increasing human landscape alterations may negatively affect golden eagle ( *Aquila chrysaetos* ) populations, calling for organized monitoring programs. Using genetic tools to monitor populations is contingent on genetic markers and methods that provide accurate genotyping from available DNA source material (e.g., feathers, blood etc.). Here, we describe a SNP panel for the golden eagle in northern Europe to determine sex, identify individuals, analyze relatedness, and genetic diversity. Samples from Sweden, Norway, Denmark, and Finland were RAD‐sequenced, and 29,361 SNP markers were ascertained in 96 samples. Markers that did not contain a single biallelic SNP or were not present in at least half of the population were excluded. These were filtered down to 219 markers with a mean minor allele frequency (MAF) of 0.36. Based on clustering performance, 95 of the 219 markers were selected. An additional marker, CHD1ZW, was added to determine sex. The final panel was successful in sexing and identifying individuals, though assessing relatedness using this panel should be done with caution. This new SNP panel constitutes a valuable resource for applications in management and ecological research.

## Graphical abstract

We describe and validate a 96 SNP panel for golden eagles in northern Europe as a tool for monitoring and future studies. We show that it can determine sex, identify individuals, and assess relatedness with great accuracy.

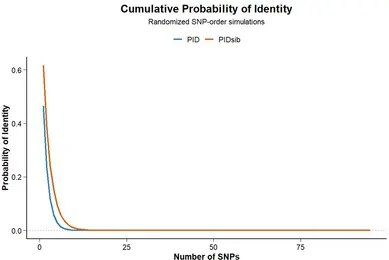

## 1 Introduction

Increasing human alterations to natural ecosystems are placing many populations of wild species at risk. In Fennoscandia (Norway, Sweden, and Finland), most of these alterations are related to intensive forestry, expansion of infrastructure, and climate change, causing rapidly changing ecosystems (Mönkkönen et al. 2022; Balotari‐Chiebáo and Byholm 2024; Strandberg et al. 2025). Apex predators are especially sensitive to such changes, as they tend to be long‐lived, have relatively low reproductive rates, and small population sizes. They often require vast areas of suitable habitat to survive and reproduce (Holt et al. 1999; McGrady et al. 2002).

The golden eagle ( *Aquila chrysaetos* ) is a large, long‐lived raptor species that reproduces slowly and has a long generation time (Steenhof et al. 1997; Karabanina et al. 2024). Golden eagles have small clutch sizes, most often only laying one or two eggs, and the young sometimes do not settle in a territory until they are around 5 years of age (Steenhof et al. 1997; Whitfield et al. 2022). Survival is also low for the first few years but increases fast until adulthood (Nygård et al. 2016; Millsap et al. 2022). This makes this species very sensitive to increased adult mortality. In Fennoscandia, the main breeding population is in the north, with a smaller breeding population in the southernmost part of Sweden and Denmark (Mullarney et al. 2010). In addition, a high density of breeding pairs of golden eagles is found on the Swedish island of Gotland (Åsbrink and Hellström 2025). The golden eagle population in Fennoscandia experienced a rapid decline and local extinctions during the early 1900s because of persecution, habitat destruction, and later on accumulation of environmental toxins, but the number of breeding pairs has steadily increased again since the 1970s, mainly due to legal protections (Watson 1992; Pohja‐Mykrä et al. 2012). In later years the golden eagle population has been stable (Saurola 2008), though breeding attempts and nesting success differ strongly between years based on prey availability and weather (Tjernberg 1983; Steenhof et al. 1997; Nyström et al. 2006; Moss et al. 2012). Despite successful recovery, populations can still suffer a decline in genetic diversity as a consequence of low population size in recent history.

Genetic studies have revealed phylogeographic structure across the golden eagle's range. Two major mitochondrial lineages have been identified: a Holarctic lineage distributed across Europe, Asia, and North America, and a Mediterranean lineage found around the Mediterranean (Nebel et al. 2015). More recently, whole‐genome analyzes have suggested that the Mediterranean lineage is more closely related to Fennoscandian populations than are populations in Japan and North America (Sato et al. 2024). Within this broader context, the genetic consequences of the historical bottleneck in Fennoscandia remain unclear. Nebel et al. (2019) did not find a bottleneck signature in northern Europe, based on microsatellite data. Similarly, genetic diversity did not differ substantially from the rest of Europe, despite historic persecution and the recent rebound (Nebel et al. 2019). This could be because of high levels of gene flow. Nebel et al. (2019) found that there were similar levels of gene flow both within Fennoscandia and between Fennoscandia and the Alps. However, Karabanina et al. (2024), who used historic samples from museums, noted a decrease in the number of haplotypes after the bottleneck, suggesting that golden eagles of the palearctic lineage of mainland Eurasia may have experienced a bottleneck. Karabanina et al. (2024) also noted that both mitochondrial and nuclear genetic variation in the Fennoscandian population was rather low compared to other parts of the Eurasian continent, in particular compared to central Asia and Caucasus, though still higher than Scotland. However, most of the samples from northern Europe in this study were from northwestern Russia, leaving the diversity in Fennoscandia uncertain. It is possible that the Fennoscandian population has indeed suffered a loss of genetic diversity as a consequence of a bottleneck. Sato et al. (2024) argue that small microsatellite panels, like the one used by Nebel et al. (2019), lack the strength to detect bottleneck patterns. Bottlenecks and the decline in genetic diversity can make populations more sensitive to environmental changes (Frankham 1995), highlighting the importance of organized monitoring programs. Resolving patterns of genetic diversity, relatedness, and connectivity therefore requires genetic tools with sufficient resolution for large‐scale monitoring.

Monitoring golden eagle populations is challenging because the species is elusive and ranges across vast areas (McGrady et al. 2002). Presently, the Fennoscandian countries have different monitoring programs for golden eagles (Saurola 2008; Hellström and Helander 2012; Gjershaug et al. 2018). These include visiting territories to document occupancy and reproductive success, and genetic monitoring. Genetic monitoring can be performed using noninvasive sampling, such as shed feathers, avoiding unnecessary stress to the animals (Schwartz et al. 2006). However, noninvasive samples may yield low DNA concentration and may be of lower quality compared to DNA extracted from tissue and blood samples (Khan et al. 2020), possibly leading to poor genotyping quality. An increasingly popular solution to this challenge is the use of Single Nucleotide Polymorphism (SNP) panels in conjunction with nanofluidic‐based genotyping platforms. Unlike widely used microsatellites, SNP panels are less sensitive to samples with low quality DNA because of the shorter target DNA sequence compared to microsatellites (Morin et al. 2004). In addition, SNP data are more suitable for comparisons of samples genotyped in different labs, allowing for increased cooperations between countries that share a population (Morin et al. 2004). However, microsatellite markers are more informative on their own compared to SNP markers, meaning more SNP markers are needed for comparable strength when analyzing genetic diversity and relatedness assessment (Morin et al. 2004). A panel composed of several SNP markers distributed across the genome therefore allows for both time and cost‐efficient genotyping of large sample sets and high‐quality genotype data despite low‐quality target DNA (Okitsu et al. 2013; Forbes et al. 2025). Genetic monitoring allows for more detailed data on eagles than observations in a territory alone, while simultaneously having the potential to be less invasive than direct handling of birds. For example; wolverines ( *Gulo gulo* ), bears ( *Ursus arctos* ), and wolves ( *Canis lupus* ) are all monitored using noninvasive genetic sampling in Norway and Sweden (Bischof et al. 2019). An SNP panel designed to identify individuals, close relatives and potential genetic structures can provide information on reproductive success, territory turnover rates, and gene flow (dispersal) for a better understanding of how this apex predator is affected by ecosystem change. Implementation of such a panel in the organized monitoring of the species could help catch early signs of distress to the population. For example, in Japan microsatellite markers has been used to study connectivity and diversity in the local golden eagle populations (Naito‐Liederbach et al. 2021). This study also emphasized the importance of continuous genetic monitoring to catch early signs of bottlenecks and decreased connectivity (Naito‐Liederbach et al. 2021).

Consequently, the development of a standardized SNP panel may facilitate coordinated genetic monitoring across Fennoscandia while providing higher resolution than traditional microsatellite markers. Here, we present an SNP panel with reliable performance across sample materials, including noninvasively collected feathers. The panel holds 96 markers ascertained in the Fennoscandian golden eagle population. We aimed to develop a panel with sufficient resolution to: (1) identify sex, (2) identify individuals, (3) assess relatedness among the individuals, (4) provide information on genetic diversity and structure, and (5) detect possible white‐tailed eagle ( *Haliaeetus albicilla* ) samples mistakenly included among the noninvasive samples.

## 2 Methods

### 2.1 Sampling and Extraction

We collected 96 blood and tissue samples for RAD‐sequencing from golden eagles from different parts of Sweden, Norway, Denmark, and Finland in cooperation with the nongovernmental organization (NGO) Golden Eagle Sweden, the Swedish Veterinary Agency (Statens Veterinärmedicinska Anstalt, SVA), and the Norwegian Institute for Nature Research (NINA) (Appendix S1). A total of 83 blood samples were taken: 58 when the eaglets were ringed before they left the nest, as part of the ecological monitoring of the golden eagle programs; 25 adult eagles were captured and sampled while being tagged for other research projects; and 13 tissue samples originated from dead birds and were provided by the SVA, who is responsible for handling the dead animals as part of monitoring projects, and the University of Agder and the NTNU University Museum in Norway (Appendix S1).

Tissue samples were stored at −18°C in 96% ethanol, and blood samples were stored dry at room temperature on FTA cards or in alcohol at 8°C in a fridge. DNA was extracted with a Qiagen QIAsymphony DSP DNA Mini Kit according to the manufacturer's protocol using a Qiagen QIAsymphony instrument. DNA concentrations were measured using a Thermo Scientific NanoDrop 2000 Spectrometer. DNA quality was visually examined by running 3 μL of the sample on a 1.5% agarose gel.

### 2.2 Sequencing and Marker Selection

**FIGURE 1:**
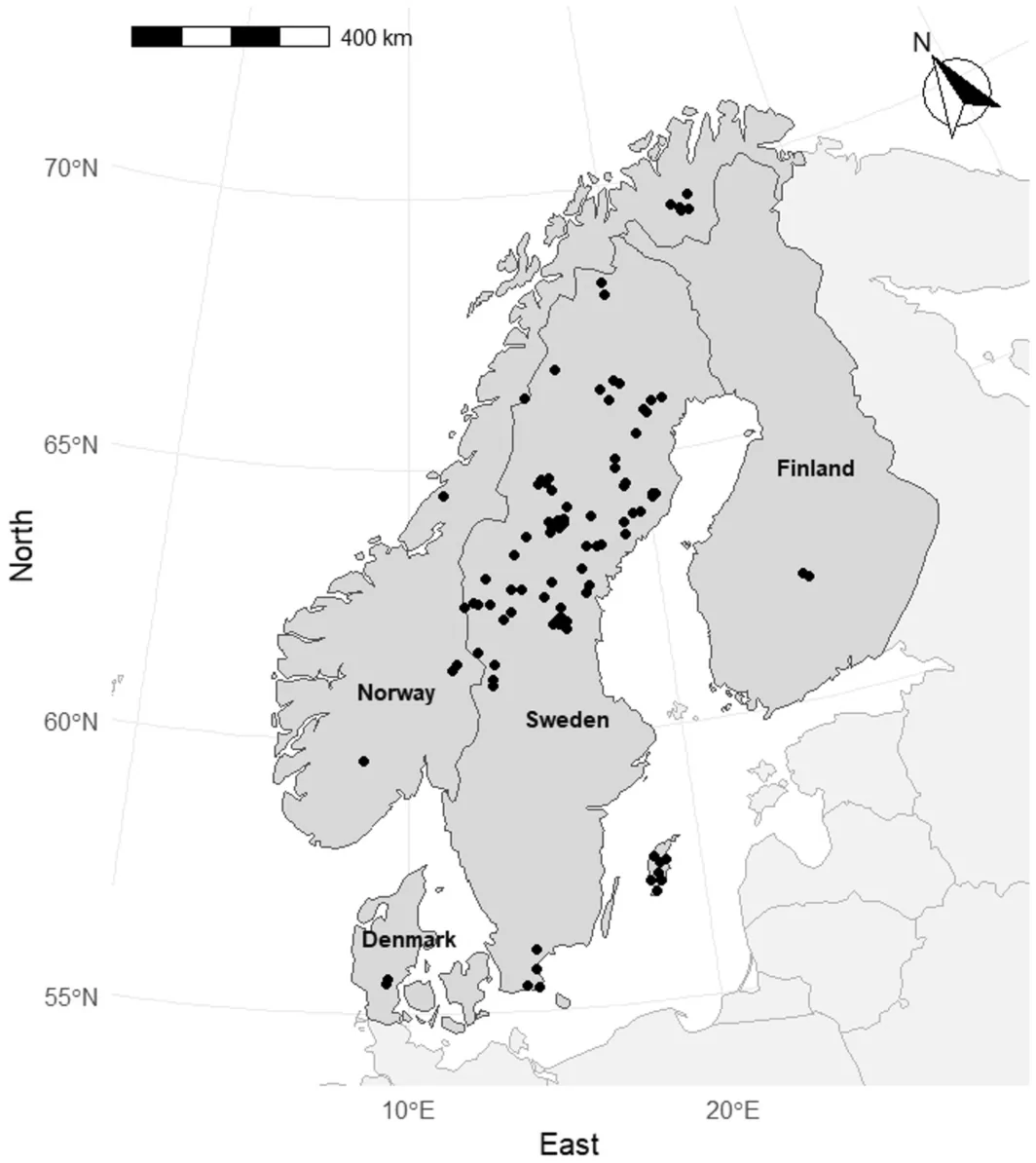
The origin of the 96 golden eagle samples used for the development of a 96‐SNP panel for the Fennoscandian golden eagle population.

The majority of these samples (*N* = 42) originated from the two northernmost counties in Sweden (Norrbotten and Västerbotten counties), 12 from central Sweden, six from southern and central Norway, which were combined with samples from western Sweden (*N* = 17) and treated as one population (*N* = 23), seven from the island Gotland, five from Finnmark in Northernmost Norway, two from Finland, and five from Denmark and Skåne county in southern Sweden combined (Figure 1). A total of 85 μL of selected sample extracts were digested at 37°C for 3 h with 5.5 μL EcoR1, and fragments between 500 and 700 base pairs were excised. To ensure proper digestion, 3 μL of the samples were again run on a 1.5% gel to visually confirm the digestion. When digestion was complete, the samples were incubated at 80°C for 5 min to inactivate the enzyme. The samples were then frozen and sent to Science for Life Laboratory (SciLifeLab) in Sweden where they were paired‐end RAD sequenced (2 × 150) on a single lane on an Illumina HiSeq 2500, set to a rapid mode. SNP screening was performed in Stacks 1.48 (Catchen et al. 2011) under default settings. Markers containing more than a bi‐allelic SNP were filtered out. Only markers with the SNP falling in the middle of the read, assuming reads of 130 base pairs and markers with a major allele frequency of ≤ 0.75 were kept. To ensure good coverage, markers had to be present in more than half of the samples (*N* ≥ 60). SNPs not in Hardy–Weinberg equilibrium (HWE) were filtered out.

### 2.3 Genotyping and Validation

Remaining candidate SNPs were screened for clustering at NINAGEN in Trondheim, Norway on 81 separate validation samples from golden eagles from Finland (*N* = 40) and Norway (*N* = 41), as well as three white‐tailed eagles ( *Haliaeetus albicilla* ) from Norway. White‐tailed eagles were included for reference, as their feathers might be mistaken for golden eagle feathers. Ten of the golden eagle samples from Norway, all molted feathers, were run twice to estimate error rates. The 41 golden eagle samples from Norway represented 41 different individuals (22 females and 19 males), as determined through microsatellite genotyping (Gjershaug et al. 2018). The validation samples from Norway represented a variety of source material, that is, blood, bone, claw, eggshell, foot pads, molted feathers, muscle tissue and pulled feathers (Appendix S2). The validation samples from Finland were all pulled feathers from nestlings. Based on clustering performance, 95 final SNPs were selected. One SNP (CHD1ZW) from Doyle et al. (2016) was added and validated for molecular sexing using known individuals. The samples were genotyped on a 96.96 dynamic array using the EP1 instrument (Standard BioTools, CA, USA) following the manufacturer's protocol and scored with the accompanying SNP genotyping analysis software.

### 2.4 Sample Material

The average percentage of missing data was calculated in RStudio (version 4.4.2) for each sample type to evaluate the genotyping amplification success of various source materials. The white‐tailed eagle samples were excluded from this analysis.

### 2.5 Sex Identification

Sex was determined based on the genotype of the sex marker CHD1ZW. The accuracy was then estimated by comparing the result with the samples of previously known sex. The sex marker was excluded from the following analyzes, leaving 95 autosomal SNP markers.

### 2.6 Individual Identification

Probability of identity (PID), probability of identity for full siblings (PIDsib) and exclusion probability, for both one and no parents known, were calculated using the R package *adegenet* (v.2.1.11). The white‐tailed eagle samples were excluded from this analysis and only the run with higher amplification success was used for the 10 samples that were run twice.

All validation samples were analyzed in the R package *allelematch* (v.2.5.2) to test the panel for identification of individuals with several samples (Galpern et al. 2012). Error rate was calculated by counting the number of mismatching alleles between the first and second run of the 10 samples that were run twice.

### 2.7 Assessing Relatedness

No pairs of samples with known relatedness were available so to test the panel for assessing relatedness we did a simulation‐based validation framework using RStudio (version 4.4.2). The white‐tailed eagle samples, and samples with amplification success below 85% were excluded. The allele frequency was estimated based on the remaining samples and used to simulate genotypes for different relatedness classes, generating 10,000 replicate pairs for each class. For each simulated pair, genomic relatedness was estimated using an allele frequency standardized genomic relatedness coefficient. For each SNP, genotypes were coded as the number of copies of the reference allele (0, 1, or 2) and centered by subtracting the expected genotype value (2*p*, where *p* is the estimated reference allele frequency). Pairwise genomic relatedness was calculated as the covariance of the standardized genotype scores divided by the expected genetic variance across loci.

Observed pairwise genomic relatedness was calculated for all possible pairs of individuals in the empirical dataset and compared with the simulated distributions to assess whether the observed variation was consistent with simulated expectations.

The ability of the SNP panel to classify relationship categories was evaluated using the simulated datasets. Because parent‐offspring and full‐sibling pairs had an almost 100% overlap, these relatedness classes were combined into a single first‐order relative category. Simulated pairs were assigned to one of three relatedness classes: unrelated, half‐sibling, or first‐order relative.

For each relatedness class, the probability density of the simulated genomic relatedness coefficients was estimated using kernel density estimation. Each simulated pair was then assigned to the relatedness class with the highest probability density at its estimated genomic relatedness coefficient. Classification performance was summarized as a confusion matrix showing the proportion of simulated pairs correctly and incorrectly assigned to each relatedness class. Overall classification accuracy was calculated as the proportion of correctly classified simulated pairs across all relatedness classes.

### 2.8 Genetic Diversity

From the genotypes of the 96 RAD‐sequenced samples we calculated observed and expected heterozygosity, inbreeding coefficient, genetic diversity, and genetic differentiation among populations using the R package *adegenet* (v.2.1.11) in RStudio (version 4.4.2).

### 2.9 White‐Tailed Eagle

The heterozygosity levels of each individual were calculated to find samples with unprecedented high and low levels of heterozygosity. In addition, the white‐tailed eagle samples were analyzed with the golden eagle samples with *allelematch* (v.2.5.2) to determine if the white‐tailed eagle samples would match with each other despite being different individuals.

## 3 Results

### 3.1 Marker Selection and Genotyping

We identified 29,361 SNP‐markers, out of which 21,959 were biallelic SNPs. After filtering, 219 candidate SNPs remained with minor allele frequencies (MAF) between 0.25 and 0.49, averaging 0.36. Out of the 219 SNPs, 189 were tested for clustering performance. Altogether, 95 SNPs and one sex marker were included in the final panel.

The DNA extracted from blood, footpad, and tissue samples all had a 100% amplification success, although sample sizes were small (Table 1). The DNA from pulled and molted feathers had amplification success of 0.962 and 0.980, respectively. Claws, eggshells, and bone samples, all of which were only represented by one sample, had comparatively low amplification success of 0.885, 0.719, and 0.615, respectively (Table 1).

**TABLE 1:** Mean amplification success per sample type with the designed 96 SNP panel for golden eagles in Fennoscandia.

Sample type	*N*	Average amplification success
Blood	6	1
Bone	1	0.615
Claw	1	0.885
Eggshell	1	0.719
Footpad	2	1
Molted feather	35	0.980
Pulled feather	43	0.962
Muscle tissue	2	1

### 3.2 Sex Identification

Out of the 81 golden eagle validation samples, 41 had been previously sexed with microsatellites. The sex marker (CHD1ZW) in the final panel called the sex correctly for 40 of the 41 samples, while the sex marker failed to amplify in the last. Thus, there were no mismatches between known sex and genotyped sex. Sexing failed to amplify in four golden eagle samples and one white‐tailed eagle sample. Sexing was successful in most sample types; the failed samples were either pulled (*N* = 3) or molted (*N* = 2) feathers. Sexing was successful and consistent in all of the samples that were run twice.

### 3.3 Individual Identification

**FIGURE 2:**
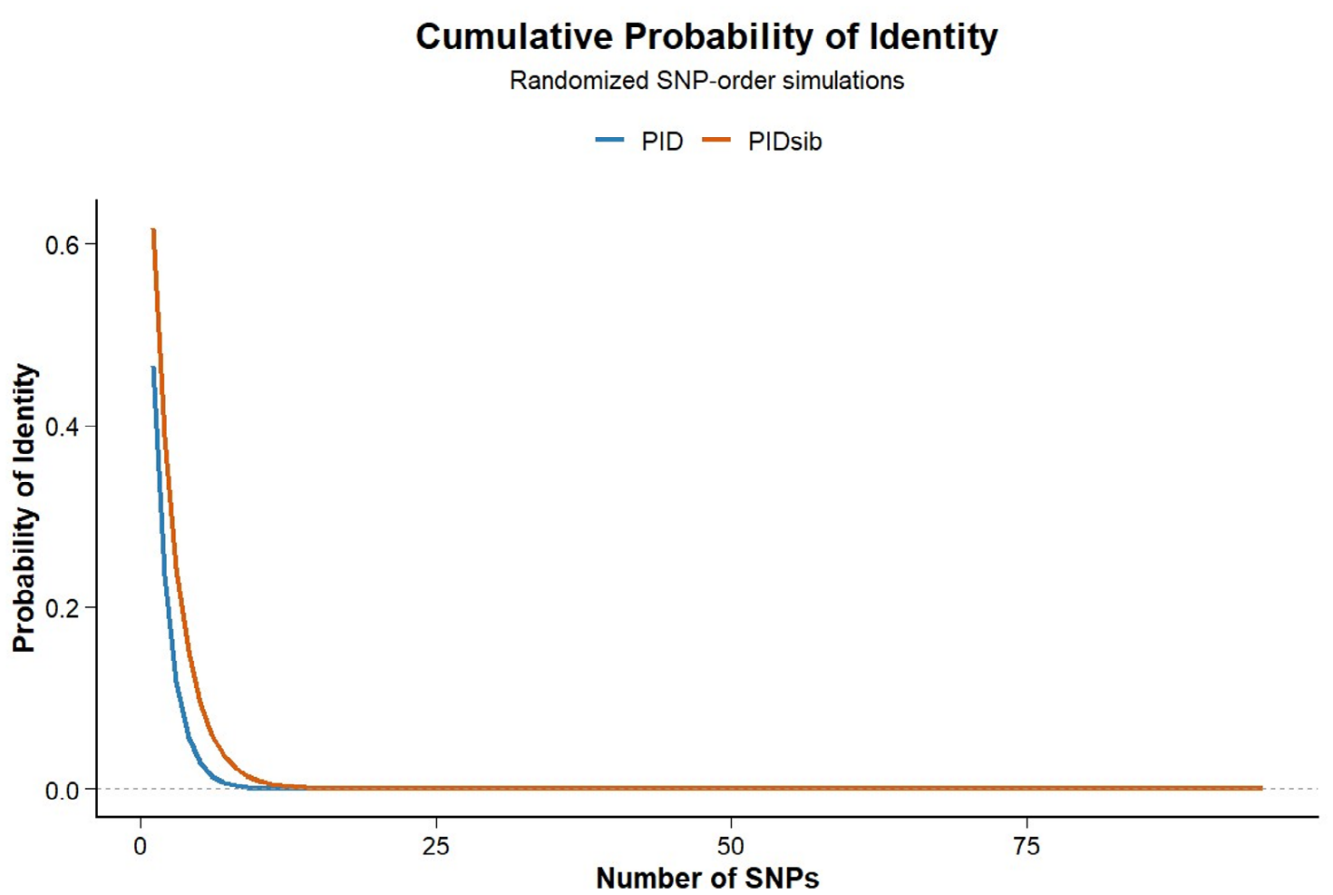
Changes in probability of identity (PID) and PIDsib depending on the number of SNP markers.

Probability of identity (PID) decreased rapidly both for unrelated individuals and siblings (PIDsib) with increasing number of SNPs until they stagnated at 15 and 25 SNPs, respectively (Figure 2). PID was 5.11 × 10^−30^, and PIDsib was 4.06 × 10^−20^ when all 95 markers were used. The exclusion probability exceeded 0.99999 for one known parent and 0.99997 for both parents unknown when the whole panel was used.

The overall error rate was low, averaging 1.979% over the 10 samples genotyped twice. Seven of these 10 molted feather samples had an error rate of 0.000. All these seven samples were genotyped with 100% success. The error rate was higher than 10%, with 11 mismatches between the two genotyping trials, in one sample only. This sample had lower amplification success, 0.990 and 0.896, than the other samples genotyped twice and was the only sample where both runs had amplification success < 1. The remaining two individuals had error rates of 1.05% with one mismatch and 7.29% with seven mismatches, respectively.

### 3.4 Assessing Relatedness

**FIGURE 3:**
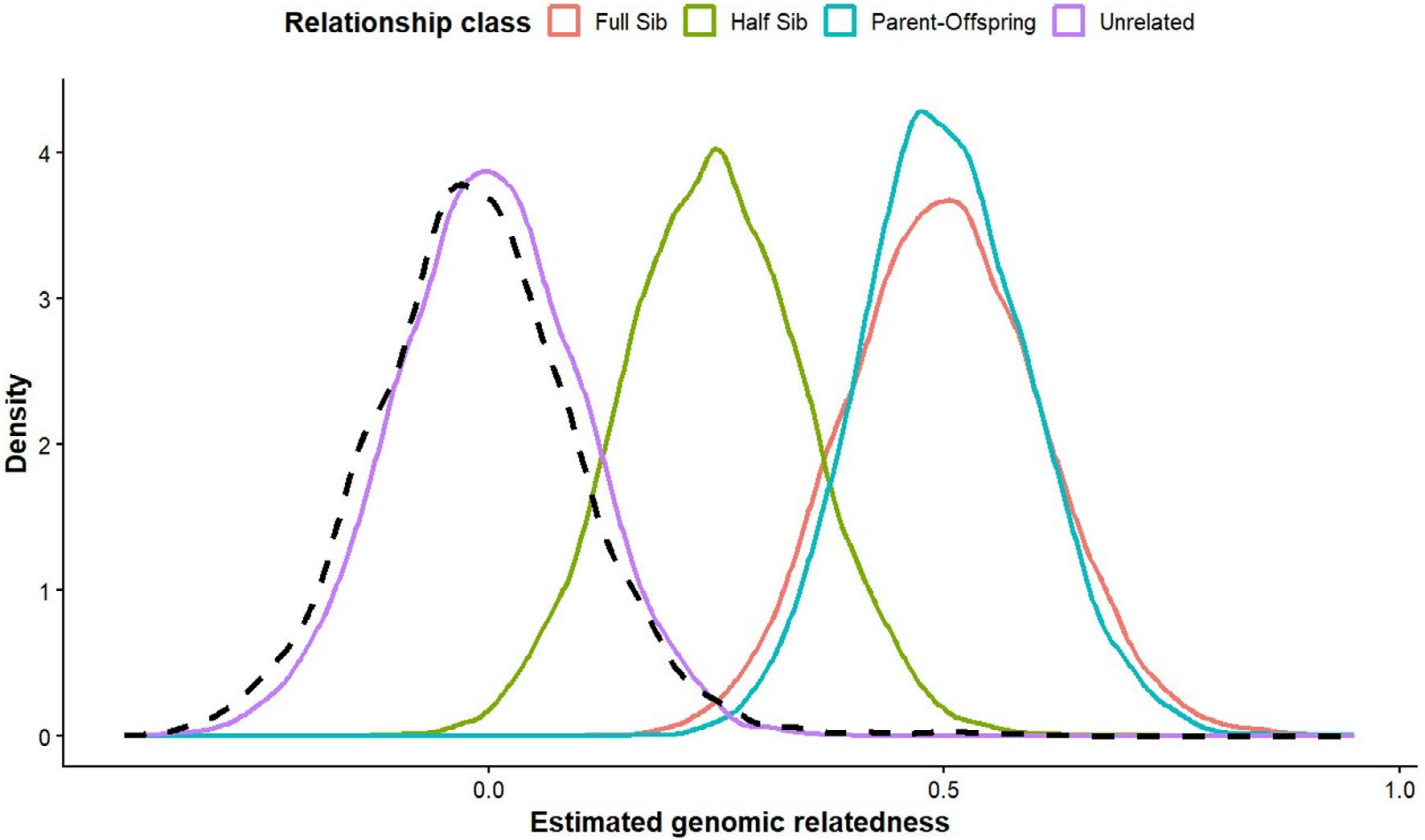
Simulated and observed genomic relatedness estimate based on allele frequencies of 95 SNP markers from 81 golden eagle samples. The dashed line is the observed relatedness estimates and the filled lines are simulations of distribution of unrelated individuals (purple), half siblings (green), parent‐offsprings (blue), and full siblings (red).

The simulation showed mean genomic relatedness coefficients at 0.000 (SD = 0.104) for unrelated pairs, 0.251 (SD = 0.102) for half‐siblings, 0.499 (SD = 0.094) for parent‐offspring pairs, and 0.502 (SD = 0.106) for full siblings. This resulted in an almost perfect overlap between full siblings and parent offspring in the genomic relatedness estimate distribution (Figure 3). The simulation showed some overlap between first‐order relatives and half siblings and similar overlap between unrelated pairs and half siblings. The simulation showed very little overlap between close relatives and unrelated pairs. Observed pairwise genomic relatedness ranged from −0.371 to 0.607 (median = −0.017, mean = −0.014). Observed pairwise genomic relatedness followed the simulated curve for unrelated pairs almost perfectly, suggesting that most samples were unrelated. The observed distribution was only slightly more variable than the simulated distribution (SD = 0.114 for the observed; SD = 0.104 for the simulated).

Based on the simulation, the panel had an overall accuracy of 86.5% when assessing relatedness based on genomic relatedness estimate. For first‐order relatives, the accuracy was 90.2% with 9.8% of pairs being incorrectly identified as half‐siblings and < 0.0% of pairs were incorrectly identified as non‐related. Similar accuracy for the non‐related pairs was 88.6% with 11.3% of pairs being incorrectly identified as half‐siblings. Less than 0.0% was incorrectly identified as first‐order relatives. The accuracy for half‐siblings was lower, with only 77.1% being correctly identified as half‐siblings. Half‐siblings were incorrectly identified as unrelated pairs in 11.4% of cases and as first‐order relatives in 11.5% of cases.

### 3.5 Genetic Diversity

Observed heterozygosity varied slightly between populations (*H*_o_ = 0.363–0.455), with generally small deviations from expected heterozygosity (*H*_e_ = 0.412–0.531) (Table 2). Most population units showed *F*_is_ values near zero, although Finland exhibited a heterozygote deficit (*F*_is_ = 0.165), while several populations showed slight heterozygote excess (e.g., Gotland: −0.083). Overall diversity was *H*_e_ = 0.449 and *H*_o_ = 0.435 (*F*_is_ = 0.031). Genetic differentiation among populations was low (*F*_st_ = 0.040; *F*_stp_ = 0.046; *D*_est_ = 0.039).

**TABLE 2:** Expected (*H*_e_) and observed (*H*_o_) heterozygosity, and inbreeding within populations (*F*_is_) in golden eagle subpopulations in Fennoscandia.

Population	*N*	*H*_e_	*H*_o_	*F*_is_
South	5	0.442	0.440	−0.014
Finland	2	0.531	0.363	0.165
Finnmark	5	0.444	0.455	−0.041
Gotland	7	0.412	0.444	−0.083
N. Sweden	42	0.459	0.455	0.003
C. Sweden	12	0.450	0.451	−0.014
Norway, W. Sweden	23	0.451	0.437	0.028
Total	96	0.449	0.435	0.031

Abbreviations: C. Sweden, Central Sweden; N. Sweden, Northern Sweden; W. Sweden, Western Sweden.

### 3.6 White‐Tailed Eagle

All three white‐tailed eagle samples had low amplification success, between 0.76 and 0.57. Two of these samples resulted in very similar genotypes with only one mismatching allele. The third sample had too much missing data for evaluation. In addition, the white‐tailed eagle samples had a heterozygosity of zero (*N* = 2) or very close to zero (0.015, *N* = 1).

## 4 Discussion

### 4.1 Genotyping Different Sample Materials

Genotyping with the designed SNP panel using DNA extracted from various sample types was successful. The highest amplification success was from blood, footpads, and tissue samples. This was expected because blood from birds is rich in DNA (red blood cells are also nucleated), and tissue samples are generally rich in high‐quality DNA when stored properly. However, the sample sizes of these tissue samples were small. Pulled and molted feathers had very good amplification success and a larger sample size than the previously mentioned sample materials. The high amplification success in feathers is likely due to blood remains inside the feather, a leftover from the development phase of the feather (Horváth et al. 2004). This suggests that the SNP panel is effective for genotyping easily collectable feather samples, irrespective of whether they have been invasively plucked or noninvasively found in the environment. The amplification success difference between pulled and molted feather samples was very small and nonsignificant (β = 0.03 ± 0.12 SE, *z* = 0.25, *p* = 0.803). Surprisingly, molted feathers even had a slightly higher mean amplification success than pulled feathers, further strengthening the argument to use noninvasively sampled feathers.

Pulled feathers as samples for genotyping still come with the advantage of knowing with certainty from which individual the sample originates, but obtaining these types of samples requires contact with the animal, which causes stress to the animal, risk of injury to both animal and handler, and is often more labor‐intensive than noninvasive sampling (Zemanova 2021). Drawing blood and plucking feathers both cause physical pain and discomfort to the animal. The remaining sample materials, eggshells, bones, and claws had relatively low amplification success. While the sample sizes of these sample materials were small, these sources are better avoided if other sample materials are available.

### 4.2 Sex Identification

The main aim for developing this panel was to identify sex and individual for yearly monitoring from a variety of sample materials collected either directly from eaglets when ringing or collected noninvasively in or around the nest. The panel demonstrated high reliability in sex determination; the single unsuccessful case was attributable to missing data, and no misidentifications were observed.

### 4.3 Identification of Individuals

In addition, low PID and PIDsib indicate that the panel is very strong in identifying individuals. This was further supported by a low error rate. Only in one of the comparisons among the same samples were the obtained genotypes not identified as originating from the same individual because of the high level of missing data in the second run. That low amplification rates correlate to genotyping error is well‐established and emphasizes the need for careful quality control, amplification thresholds, and sample pruning to avoid the inclusion of spurious data or individual genotypes. The high exclusion probability for both one and no parents known further suggests that the panel has a very low risk of incorrectly assigning parentage if genotypes are appropriately quality controlled.

Low error rate and high amplification success have been described as one of the strengths of SNP panels (Campbell and Narum 2008; Thavornkanlapachai et al. 2024). However, genotyping of noninvasive samples is inherently error prone, especially when using pre‐spotted SNP chips (Ekblom et al. 2018). SNP genotyping of noninvasive samples therefore requires careful selection of chemistry and platform, where liquid‐based systems are superior. By using such a system, our error rate for samples with 100% amplification success was zero. However, for samples with missing data above 2%, replicates showed elevated error rates. This is because such samples also hold errors where heterozygote loci have lost one allele, causing them to appear homozygote. Under the assumption of dropout homogeneity, the probability of an allele dropping out is the square root of the fraction of missing loci. By using this number and the individual heterozygosity, the expected number of genotype errors can be estimated. For samples with 2% missing data, the expected number of genotyping errors is roughly six. Given the standing genetic variation in the population, this error rate is not affecting our ability to reliably identify individuals. In comparison, Kylmänen et al. (2023) estimated an error rate of 6.21% with a microsatellite panel for Finnish golden eagles, and even after removing poorly amplified samples, the error rate remained higher (2.4%) than estimated with the SNP panel here. Note that locus level genotyping error, as used in Kylmänen et al. (2023), is different from individual level genotyping error. Even when genotyping error is low, the compounded effect across loci is important to consider. However, small sample sizes naturally come with larger confidence intervals, and our small sample size for analysis of error rate could be more inflated than for the larger sample size (*N* = 372) used by Kylmänen et al. (2023). Similarly, our overall amplification success was 99.19% compared to 84% in Kylmänen et al. (2023) further demonstrating the usefulness of SNP panels when working with noninvasive samples.

### 4.4 Assessing Relatedness

The simulation showed, as expected, two genomic relatedness estimates peaking at 0.5 for close relatives (full siblings and parent‐offspring), one peak at 0.25 for half siblings, and one peak at 0 for unrelated pairs. However, the simulated distributions showed an overlap between half siblings and unrelated pairs, and half siblings and close relatives. There was, however, almost no overlap between close relatives and unrelated pairs. Similarly, the simulation did identify first‐order relatives and non‐relatives correctly consistently, in about 90% of the pairs. Half‐siblings were, however, identified correctly in 77% of the pairs. This indicates that the panel is powerful enough to distinguish between unrelated pairs and close relatives but struggles to distinguish half siblings from unrelated pairs and close relatives. These are similar results to Glaubitz et al. (2003) who found that 100 SNPs are only able to distinguish close relatives from non‐related individuals but cannot be used to find half siblings reliably. With the overall accuracy of the panel being at 86.5%, this panel can assess relatedness to some extent but should be done with caution.

### 4.5 Genetic Diversity

The difference between expected and observed heterozygosity was generally low and very similar between populations despite different population sizes indicating similar genetic diversity among the regions. The exception was the Finnish population which had much lower heterozygosity than expected (*H*_o_ = 0.363 and *H*_e_ = 0.531). This is most likely explained by the very small sample size of two from Finland rather than a heterozygosity deficit, as Kylmänen et al. (2023), who had a much larger sample set, found no sign of heterozygosity deficit with microsatellites in Finland.

The low *F*_is_ values in each population and the total population suggest very low inbreeding. Finland was the outlier but is most likely explained by the very small sample size. Again, Kylmänen et al. (2023) who used a much larger sample set, found little support for widespread inbreeding of golden eagles in Finland. Surprisingly Gotland did not show higher inbreeding coefficient, or lower heterozygosity than the northern mainland populations. Other island populations like Scotland and Japan have shown a lower diversity than mainland Europe (Sato et al. 2024). It is possible that Gotland, despite being an island, is located in close enough proximity to the mainland to exchange geneflow despite appearing isolated on the map. However, Gotland also had a quite small sample size (*N* = 7), so these results should be interpreted with caution. Another explanation could be that the relatively low number of SNPs used here and the low number of microsatellites in Kylmänen et al. (2023) lack the power to detect potential inbreeding. Kardos et al. (2016) pointed out that many previous studies have had low precision when calculating inbreeding because they used too few microsatellite markers. While we use more markers than, for example, Kylmänen et al. (2023) SNP markers are less informative than microsatellites, suggesting more markers are needed to accurately estimate inbreeding.

The low *F*_st_ suggests low differentiation between populations. This is strengthened by Nebel et al. (2019) who found geneflow between Norway and Finland–Estonia at about the same rate as between northern and southern Europe. In addition, most of the breeding population of golden eagles in Fennoscandia is connected, continuously stretching across in land and mountainous areas of Norway and Sweden and across to the northern parts of Finland. It is also possible that Gotland and the southern population still exchange geneflow with the northern populations despite appearing isolated on the map.

### 4.6 Detection of White‐Tailed Eagle Samples

The panel was successful in detecting white‐tailed eagle samples. Two of the genotyped white‐tailed eagles were identified as the same individual by the R package *allelematch* (2.5.2) despite being different individuals. The third white‐tailed eagle was not identified as the same individual by *allelematch* (2.5.2) because of an excess of missing data, but when manually checking the genotypes of that individual it was near identical to the other 2 white‐tailed eagles when disregarding the missing data, only differing from one of the other samples at one marker. Samples mistakenly collected from white‐tailed eagles will result in relatively high amounts of missing data as well as very low heterozygosity levels. The missing data in the white‐tailed eagle genotypes likely stem from either low sample quality or absence of target sequences in this species. The low observed heterozygosity is common when applying an SNP panel for a species it was not designed for (Fountain et al. 2021). This highlights the importance of filtering out samples with low genotyping amplification success and too low heterozygosity before any analyzes, as they are likely to represent nontarget species.

## 5 Conclusions

When studying wild populations, noninvasive samples like hair, fecal, or shed feathers are good sources for DNA as they can be sampled with minimal stress to the animal (Khan et al. 2020). Microsatellites have been widely used for this purpose (Putman and Carbone 2014). However, noninvasive samples often yield low concentration and poor‐quality DNA (Khan et al. 2020). Because of the shorter target sequence in the DNA, SNP markers are less sensitive to poor quality DNA (Morin et al. 2004), potentially making them the better candidate for monitoring of species that are sensitive to human disturbance. In addition, when monitoring populations that are shared between countries, like golden eagles in Fennoscandia, genotyping data from SNP panels are more suitable than microsatellites for comparison between labs, allowing for the creation of larger databases and extensive cooperations between countries (Morin et al. 2004). The main disadvantage of SNPs is that each marker is less informative because of the number of alleles; each SNP only has two while microsatellites have several (Morin et al. 2004). However, when increasing the number of SNPs, the power of the entire panel to detect diversity and relatedness increases (Morin et al. 2004).

The new SNP panel designed for monitoring the golden eagle population in Fennoscandia proved successful in providing high‐quality data from several different sample materials, including noninvasively collected shed feathers. It can be used to determine sex, identify individuals, and infer close relatives with descent accuracy, though it struggles with half‐siblings and pinpoint samples that likely originate from other raptor species mistakenly sampled. This SNP panel shows great potential for routine monitoring and ecological studies of the Fennoscandian golden eagle population.

## Author Contributions

**Benjamin Lindberg:** writing – original draft (lead), writing – review and editing (equal). **Oddmund Kleven:** data curation (equal), validation (equal), writing – review and editing (equal). **Laura Kvist:** data curation (equal), supervision (equal), writing – review and editing (equal). **Anita Norman:** data curation (equal), writing – review and editing (equal). **Helena Köningsson:** data curation (equal), supervision (equal). **Karl‐Otto Jacobsen:** data curation (equal), writing – review and editing (equal). **Per‐Olof Nilsson:** data curation (equal), writing – review and editing (equal). **Navinder J. Singh:** funding acquisition (equal), supervision (lead), writing – review and editing (equal). **Göran Spong:** funding acquisition (equal), supervision (equal), writing – review and editing (equal).

## Funding

This work was supported by OX2. Interreg Aurora, 20366483. Fortums Stiftelse. Vattenfall. Statkraft. Naturvårdsverket.

## Conflicts of Interest

The authors declare no conflicts of interest.

## Supporting information


**Appendix S1:** Samples that the 96.96 SNP panel for golden eagles in Fennoscandia is based on. Abbreviations under “population” are defined as follows: Northern Sweden (N. Sweden), Central Sweden (C. Sweden), Western Sweden (W. Sweden), Southern Sweden and Denmark (South). Juveniles are abbreviated as “Juv” and adults as “Ad”.
**Appendix S2:** Samples used for validation of the 96.96 SNP panel for golden eagles in Fennoscandia. Red highlights are samples run twice for error rate calculation.

## Data Availability

The data is available on Dryad (https://doi.org/10.5061/dryad.mpg4f4rgg) with the exception of locational metadata because of the golden eagle's protective status in the region.
