# Supplementary material for: A Genetic Tool for Non‐Invasive Monitoring of Fennoscandian Golden Eagles (Aquila chrysaetos)
Source: Ecol Evol. 2026 Sep 20;16(9):e74386. doi: 10.1002/ece3.74386 (PMC13590845; doi:10.1002/ece3.74386)
Supplement: Supplementary file 1 [file ECE3-16-e74386-s001.docx]

**Appendix**

Appendix S1 Samples that the 96.96 SNP panel for golden eagles in Fennoscandia is based on. Abbreviations under “population” are defined as follow: Northern Sweden (N. Sweden), Central Sweden (C. Sweden), Western Sweden (W. Sweden), Southern Sweden and Denmark (South). Juveniles are abbreviated as “Juv” and adults as “Ad”.

| **DNA ID** | **Species** | **Age sampled** | **Sampling date** | **Sample material** | **Country/Region** | **Population** |
| --- | --- | --- | --- | --- | --- | --- |
| **Ac001** | Golden Eagle | Juv | 2011-06-28 | Blood | Sweden (mainland) | N. Sweden |
| **Ac004** | Golden Eagle | Juv | 2010-11-05 | Blood | Sweden (mainland) | N. Sweden |
| **Ac006** | Golden Eagle | Ad | 2010-10-19 | Blood | Sweden (mainland) | N. Sweden |
| **Ac007** | Golden Eagle | Ad | 2010-10-08 | Blood | Sweden (mainland) | N. Sweden |
| **Ac008** | Golden Eagle | Ad | 2010-10-13 | Blood | Sweden (mainland) | N. Sweden |
| **Ac009** | Golden Eagle | Ad |  | Blood | Sweden (mainland) | N. Sweden |
| **Ac010** | Golden Eagle | Juv | 2010-10-10 | Blood | Sweden (mainland) | N. Sweden |
| **Ac011** | Golden Eagle | Juv | 2010-11-06 | Blood | Sweden (mainland) | N. Sweden |
| **Ac013** | Golden Eagle | Juv | 2011-09-29 | Blood | Sweden (mainland) | N. Sweden |
| **Ac019** | Golden Eagle | Ad | 2011-10-09 | Blood | Sweden (mainland) | N. Sweden |
| **Ac023** | Golden Eagle | Ad | 2011-10-16 | Blood | Sweden (mainland) | N. Sweden |
| **Ac025** | Golden Eagle | Ad | 2011-10-20 | Blood | Sweden (mainland) | N. Sweden |
| **Ac027** | Golden Eagle | Ad | 2011-10-31 | Blood | Sweden (mainland) | N. Sweden |
| **Ac028** | Golden Eagle | Juv | 2011-10-02 | Blood | Sweden (mainland) | C. Sweden |
| **Ac029** | Golden Eagle | Juv | 2011-10-02 | Blood | Sweden (mainland) | C. Sweden |
| **Ac031** | Golden Eagle | Ad | 2011-10-07 | Blood | Sweden (mainland) | C. Sweden |
| **Ac034** | Golden Eagle | Juv | 2008 | Blood | Sweden (mainland) | N. Sweden |
| **Ac035** | Golden Eagle | Juv | 2008 | Blood | Sweden (mainland) | N. Sweden |
| **Ac036** | Golden Eagle | Juv | 2012-06-19 | Blood | Sweden (mainland) | N. Sweden |
| **Ac040** | Golden Eagle | Juv | 2008 | Blood | Sweden (mainland) | N. Sweden |
| **Ac041** | Golden Eagle | Juv | 2008 | Blood | Sweden (mainland) | N. Sweden |
| **Ac042** | Golden Eagle | Juv | 2010-06-19 | Blood | Sweden (mainland) | N. Sweden |
| **Ac046** | Golden Eagle | Juv | 2011 | Blood | Sweden (mainland) | N. Sweden |
| **Ac048** | Golden Eagle | Juv | 2008 | Blood | Sweden (mainland) | N. Sweden |
| **Ac049** | Golden Eagle | Ad |  | Blood | Sweden (mainland) | Norway, W. Sweden |
| **Ac050** | Golden Eagle | Juv |  | Blood | Sweden (mainland) | Norway, W. Sweden |
| **Ac051** | Golden Eagle | Juv |  | Blood | Sweden (mainland) | Norway, W. Sweden |
| **Ac052** | Golden Eagle | Juv |  | Blood | Sweden (mainland) | Norway, W. Sweden |
| **Ac053** | Golden Eagle | Juv |  | Blood | Sweden (mainland) | Norway, W. Sweden |
| **Ac054** | Golden Eagle | Juv |  | Blood | Sweden (mainland) | C. Sweden |
| **Ac055** | Golden Eagle | Juv |  | Blood | Sweden (mainland) | Norway, W. Sweden |
| **Ac056** | Golden Eagle | Juv |  | Blood | Sweden (mainland) | N. Sweden |
| **Ac057** | Golden Eagle | Juv | 2014-07-10 | Blood | Sweden (mainland) | N. Sweden |
| **Ac058** | Golden Eagle | Juv |  | Blood | Sweden (mainland) | N. Sweden |
| **Ac059** | Golden Eagle | Juv | 2014-07-10 | Blood | Sweden (mainland) | N. Sweden |
| **Ac060** | Golden Eagle | Juv | 2014-07-11 | Blood | Sweden (mainland) | N. Sweden |
| **Ac062** | Golden Eagle | Ad |  | Blood | Sweden (mainland) | N. Sweden |
| **Ac063** | Golden Eagle | Juv |  | Blood | Sweden (mainland) | N. Sweden |
| **Ac064** | Golden Eagle | Juv |  | Blood | Sweden (mainland) | N. Sweden |
| **Ac065** | Golden Eagle | Juv |  | Blood | Sweden (Gotland) | Gotland |
| **Ac066** | Golden Eagle | Juv |  | Blood | Sweden (Gotland) | Gotland |
| **Ac067** | Golden Eagle | Juv |  | Blood | Sweden (Gotland) | Gotland |
| **Ac068** | Golden Eagle | Juv |  | Blood | Sweden (mainland) | Norway, W. Sweden |
| **Ac069** | Golden Eagle | Ad |  | Blood | Sweden (mainland) | Norway, W. Sweden |
| **Ac070** | Golden Eagle | Ad |  | Blood | Sweden (mainland) | N. Sweden |
| **Ac071** | Golden Eagle | Juv | 2014-07-11 | Blood | Sweden (mainland) | N. Sweden |
| **Ac072** | Golden Eagle | Juv | 2014-07-14 | Blood | Sweden (mainland) | N. Sweden |
| **Ac073** | Golden Eagle | Juv | 2014-07-14 | Blood | Sweden (mainland) | N. Sweden |
| **Ac074** | Golden Eagle | Juv |  | Blood | Sweden (mainland) | N. Sweden |
| **Ac075** | Golden Eagle | Juv |  | Blood | Sweden (mainland) | N. Sweden |
| **Ac077** | Golden Eagle | Ad |  | Blood | Sweden (mainland) | C. Sweden |
| **Ac078** | Golden Eagle | Juv | 2004-06-15 | Blood | Sweden (mainland) | C. Sweden |
| **Ac079** | Golden Eagle | Ad |  | Blood | Sweden (mainland) | C. Sweden |
| **Ac080** | Golden Eagle | Juv |  | Blood | Sweden (mainland) | C. Sweden |
| **Ac081** | Golden Eagle | Juv |  | Blood | Sweden (mainland) | Norway, W. Sweden |
| **Ac082** | Golden Eagle | Juv |  | Blood | Sweden (mainland) | C. Sweden |
| **Ac083** | Golden Eagle | Juv |  | Blood | Sweden (mainland) | C. Sweden |
| **Ac084** | Golden Eagle | Juv |  | Blood | Sweden (mainland) | C. Sweden |
| **Ac085** | Golden Eagle | Juv |  | Blood | Sweden (mainland) | C. Sweden |
| **Ac086** | Golden Eagle | Juv |  | Blood | Sweden (mainland) | Norway, W. Sweden |
| **Ac087** | Golden Eagle | Juv |  | Blood | Sweden (mainland) | Norway, W. Sweden |
| **Ac088** | Golden Eagle | Juv |  | Blood | Sweden (mainland) | Norway, W. Sweden |
| **Ac089** | Golden Eagle | Juv |  | Blood | Sweden (mainland) | Norway, W. Sweden |
| **Ac090** | Golden Eagle | Juv |  | Blood | Sweden (mainland) | Norway, W. Sweden |
| **Ac091** | Golden Eagle | Juv |  | Blood | Sweden (mainland) | Norway, W. Sweden |
| **Ac092** | Golden Eagle | Juv |  | Blood | Sweden (mainland) | Norway, W. Sweden |
| **Ac093** | Golden Eagle | Juv |  | Blood | Sweden (mainland) | Norway, W. Sweden |
| **Ac094** | Golden Eagle | Ad |  | Blood | Finland | Finland |
| **Ac095** | Golden Eagle | Ad |  | Blood | Finland | Finland |
| **Ac096** | Golden Eagle | Ad |  | Blood | Sweden (mainland) | N. Sweden |
| **Ac097** | Golden Eagle | Ad |  | Blood | Sweden (mainland) | N. Sweden |
| **Ac098** | Golden Eagle | Ad |  | Blood | Sweden (mainland) | N. Sweden |
| **Ac102** | Golden Eagle | Ad |  | Blood | Sweden (mainland) | N. Sweden |
| **Ac104** | Golden Eagle | Ad |  | Blood | Sweden (mainland) | N. Sweden |
| **Ac106** | Golden Eagle | Juv |  | Blood | Sweden (mainland) | N. Sweden |
| **Ac109** | Golden Eagle |  |  | Tissue | Sweden (mainland) | South |
| **Ac110** | Golden Eagle |  |  | Tissue | Sweden (mainland) | South |
| **Ac111** | Golden Eagle |  |  | Tissue | Sweden (mainland) | South |
| **Ac112** | Golden Eagle | Ad |  | Tissue | Norway | Norway, W. Sweden |
| **Ac113** | Golden Eagle | Ad |  | Tissue | Norway | Norway, W. Sweden |
| **Ac115** | Golden Eagle |  |  | Blood | Denmark | South |
| **Ac116** | Golden Eagle |  |  | Blood | Denmark | South |
| **Ac117** | Golden Eagle |  |  | Tissue | Sweden (Gotland) | Gotland |
| **Ac118** | Golden Eagle |  |  | Tissue | Sweden (Gotland) | Gotland |
| **Ac119** | Golden Eagle |  |  | Tissue | Sweden (Gotland) | Gotland |
| **Ac120** | Golden Eagle |  |  | Tissue | Sweden (Gotland) | Gotland |
| **Ac121** | Golden Eagle | Ad |  | Tissue | Norway | Norway, W. Sweden |
| **Ac122** | Golden Eagle | Juv |  | Tissue | Norway | Norway, W. Sweden |
| **Ac123** | Golden Eagle | Ad |  | Tissue | Norway | Norway, W. Sweden |
| **Ac124** | Golden Eagle | Ad |  | Tissue | Norway | Norway, W. Sweden |
| **Ac125** | Golden Eagle | Juv |  | Blood | Norway | Finnmark |
| **Ac126** | Golden Eagle | Juv |  | Blood | Norway | Finnmark |
| **Ac127** | Golden Eagle | Juv |  | Blood | Norway | Finnmark |
| **Ac128** | Golden Eagle | Juv |  | Blood | Norway | Finnmark |
| **Ac129** | Golden Eagle | Juv |  | Blood | Norway | Finnmark |
| **Ac130** | Golden Eagle | Ad |  | Blood | Sweden (mainland) | N. Sweden |
|  |  |  |  |  |  |  |

Appendix S2 Samples used for validation of the 96.96 SNP panel for golden eagles in Fennoscandia. Red highlights are samples run twice for error rate calculation.

| **Sample ID** | **Species** | **Individual** | **sexSTRs** | **sexSNP** | **Country sample origin** | **Sample material** |
| --- | --- | --- | --- | --- | --- | --- |
| 18 | golden eagle | Aqc0045 | Male | Male | Norway | Eggshell |
| 195 | golden eagle | Aqc0093 | Male | Male | Norway | Molted feather |
| 197 | golden eagle | Aqc0095 | Female | Female | Norway | Footpad |
| 202 | golden eagle | Aqc0097 | Female | Female | Norway | Footpad |
| 399 | golden eagle | Aqc0149 | Male | Male | Norway | Molted feather |
| 407 | golden eagle | Aqc0077 | Female | NA | Norway | Molted feather |
| 409 | golden eagle | Aqc0152 | Female | Female | Norway | Muscle tissue |
| 410 | golden eagle | Aqc0153 | Female | Female | Norway | Muscle tissue |
| 448 | golden eagle | Aqc0160 | Female | Female | Norway | Blood |
| 449 | golden eagle | Aqc0161 | Male | Male | Norway | Blood |
| 450 | golden eagle | Aqc0162 | Male | Male | Norway | Blood |
| 481 | golden eagle | Aqc0136 | Female | Female | Norway | Molted feather |
| 483 | golden eagle | Aqc0175 | Female | Female | Norway | Molted feather |
| 484 | golden eagle | Aqc0176 | Female | Female | Norway | Molted feather |
| 534 | golden eagle | Aqc0178 | Male | Male | Norway | Claw |
| 569 | golden eagle | Aqc0192 | Male | Male | Norway | Molted feather |
| 569,2 | golden eagle | Aqc0192 | Male | Male | Norway | Molted feather |
| 570 | golden eagle | Aqc0003 | Female | Female | Norway | Molted feather |
| 570,2 | golden eagle | Aqc0003 | Female | Female | Norway | Molted feather |
| 572 | golden eagle | Aqc0193 | Male | Male | Norway | Molted feather |
| 572,2 | golden eagle | Aqc0193 | Male | Male | Norway | Molted feather |
| 573 | golden eagle | Aqc0041 | Male | Male | Norway | Molted feather |
| 573,2 | golden eagle | Aqc0041 | Male | Male | Norway | Molted feather |
| 574 | golden eagle | Aqc0016 | Male | Male | Norway | Molted feather |
| 574,2 | golden eagle | Aqc0016 | Male | Male | Norway | Molted feather |
| 593 | golden eagle | Aqc0195 | Male | Male | Norway | Bone |
| 601 | golden eagle | Aqc0196 | Male | Male | Norway | Blood |
| 603 | golden eagle | Aqc0198 | Male | Male | Norway | Blood |
| 605 | golden eagle | Aqc0199 | Female | Female | Norway | Molted feather |
| 608 | golden eagle | Aqc0201 | Female | Female | Norway | Molted feather |
| 617 | golden eagle | Aqc0202 | Male | Male | Norway | Molted feather |
| 622 | golden eagle | Aqc0203 | Female | Female | Norway | Molted feather |
| 632 | golden eagle | Aqc0036 | Female | Female | Norway | Molted feather |
| 632,2 | golden eagle | Aqc0036 | Female | Female | Norway | Molted feather |
| 649 | golden eagle | Aqc0209 | Female | Female | Norway | Molted feather |
| 649,2 | golden eagle | Aqc0209 | Female | Female | Norway | Molted feather |
| 650 | golden eagle | Aqc0211 | Female | Female | Norway | Pulled feather |
| 651 | golden eagle | Aqc0212 | Female | Female | Norway | Pulled feather |
| 686 | golden eagle | Aqc0125 | Male | Male | Norway | Molted feather |
| 697 | golden eagle | Aqc0223 | Female | Female | Norway | Molted feather |
| 704 | golden eagle | Aqc0217 | Male | Male | Norway | Pulled feather |
| 705 | golden eagle | Aqc0220 | Female | Female | Norway | Molted feather |
| 707 | golden eagle | Aqc0221 | Female | Female | Norway | Molted feather |
| 708 | golden eagle | Aqc0222 | Male | Male | Norway | Molted feather |
| 710 | golden eagle | Aqc0224 | Female | Female | Norway | Molted feather |
| 710,2 | golden eagle | Aqc0224 | Female | Female | Norway | Molted feather |
| 715 | golden eagle | Aqc0225 | Female | Female | Norway | Molted feather |
| 715,2 | golden eagle | Aqc0225 | Female | Female | Norway | Molted feather |
| 717 | golden eagle | Aqc0226 | Male | Male | Norway | Molted feather |
| 717,2 | golden eagle | Aqc0226 | Male | Male | Norway | Molted feather |
| 734 | white-tailed eagle | Havorn0016 | Female | NA | Norway | Molted feather |
| 738 | white-tailed eagle | Havorn0012 | Male | Male | Norway | Molted feather |
| 739 | golden eagle | Aqc0227 | Male | Male | Norway | Blood |
| 740 | white-tailed eagle | Havorn0017 | Male | Male | Norway | Blood |
| AC1034 | golden eagle | Aqc0283 | NA | Male | Finland | Pulled feather |
| AC1038 | golden eagle | Aqc0284 | NA | Male | Finland | Pulled feather |
| AC1140 | golden eagle | Aqc0285 | NA | Male | Finland | Pulled feather |
| AC1143 | golden eagle | Aqc0286 | NA | Female | Finland | Pulled feather |
| AC1207 | golden eagle | Aqc0287 | NA | Female | Finland | Pulled feather |
| AC1211 | golden eagle | Aqc0288 | NA | Female | Finland | Pulled feather |
| AC202 | golden eagle | Aqc0289 | NA | Male | Finland | Pulled feather |
| AC204 | golden eagle | Aqc0301 | NA | Female | Finland | Pulled feather |
| AC205 | golden eagle | Aqc0302 | NA | Female | Finland | Pulled feather |
| AC233 | golden eagle | Aqc0303 | NA | Male | Finland | Pulled feather |
| AC290 | golden eagle | Aqc0304 | NA | Male | Finland | Pulled feather |
| AC296 | golden eagle | Aqc0290 | NA | Female | Finland | Pulled feather |
| AC298 | golden eagle | Aqc0291 | NA | Male | Finland | Pulled feather |
| AC300 | golden eagle | Aqc0292 | NA | Male | Finland | Pulled feather |
| AC357 | golden eagle | Aqc0305 | NA | Female | Finland | Pulled feather |
| AC372 | golden eagle | Aqc0306 | NA | Male | Finland | Pulled feather |
| AC374 | golden eagle | NA | NA | NA | Finland | Pulled feather |
| AC378 | golden eagle | Aqc0307 | NA | Male | Finland | Pulled feather |
| AC438 | golden eagle | Aqc0308 | NA | Male | Finland | Pulled feather |
| AC528 | golden eagle | Aqc0293 | NA | Female | Finland | Pulled feather |
| AC547 | golden eagle | Aqc0309 | NA | Female | Finland | Pulled feather |
| AC563 | golden eagle | Aqc0294 | NA | Female | Finland | Pulled feather |
| AC581 | golden eagle | Aqc0295 | NA | Male | Finland | Pulled feather |
| AC585 | golden eagle | Aqc0296 | NA | Male | Finland | Pulled feather |
| AC738 | golden eagle | Aqc0310 | NA | Male | Finland | Pulled feather |
| AC812 | golden eagle | Aqc0311 | NA | Female | Finland | Pulled feather |
| AC875 | golden eagle | Aqc0312 | NA | Female | Finland | Pulled feather |
| AC879 | golden eagle | Aqc0297 | NA | Female | Finland | Pulled feather |
| AC880 | golden eagle | Aqc0298 | NA | Male | Finland | Pulled feather |
| AC882 | golden eagle | Aqc0299 | NA | Male | Finland | Pulled feather |
| AC883 | golden eagle | Aqc0245 | NA | Female | Finland | Pulled feather |
| AC885 | golden eagle | Aqc0300 | NA | Female | Finland | Pulled feather |
| AC896 | golden eagle | Aqc0313 | NA | Female | Finland | Pulled feather |
| AC909 | golden eagle | Aqc0314 | NA | Female | Finland | Pulled feather |
| AC913 | golden eagle | NA | NA | NA | Finland | Pulled feather |
| AC962 | golden eagle | Aqc0315 | NA | Female | Finland | Pulled feather |
| AC970 | golden eagle | Aqc0316 | NA | Male | Finland | Pulled feather |
| AC976 | golden eagle | Aqc0317 | NA | Female | Finland | Pulled feather |
| AC989 | golden eagle | NA | NA | NA | Finland | Pulled feather |
| AC995 | golden eagle | Aqc0318 | NA | Female | Finland | Pulled feather |
